# Lateral Approach Circumstomal Open Retrorectus Mesh Repair with Transversus Abdominis Release and Three-Point Stomal Pexy for an Acute-on-Chronic Complex Parastomal Hernia Causing High-Grade Gastric Outlet Obstruction: A Case Report

**DOI:** 10.3390/jcm15135193

**Published:** 2026-07-02

**Authors:** Khang Duy Ricky Le, Leon Yeung, Eleni Baird-Gunning, David Lloyd

**Affiliations:** 1 Department of Surgery, Launceston General Hospital, Launceston, TAS 7250, Australia; 2 School of Medicine, Faculty of Health, Deakin University, Geelong, VIC 3220, Australia; 3 Department of Medical Education, Faculty of Medicine, Dentistry and Health Sciences, The University of Melbourne, Melbourne, VIC 3010, Australia

**Keywords:** parastomal hernia, hernia repair, herniorrhaphy, ventral hernia, mesh repair

## Abstract

Acute parastomal hernia containing the stomach with subsequent mechanical gastric outlet obstruction is an exceedingly rare phenomenon given the anatomical fixation of the stomach. Herein, we present the case of an 81-year-old female with a history of a Hartmann’s procedure who developed acute vomiting. She was found to have herniation of the stomach into the parastomal hernia sac with tight angulation of the gastroduodenal junction and, therefore, gastric outlet obstruction. Following decompression with a nasogastric tube, we report an open approach with circumstomal mesh for acute emergent repair.

## 1. Background

Stomach herniation into a parastomal hernia with mechanical gastric outlet obstruction is exceedingly rare and scarcely reported in the literature. The approach to repair of these hernias remains an area of debate, with a paucity of evidence related to the best approach to timing, mesh placement and overall method of repair. This case highlights a novel approach to the emergent repair of a gastric parastomal hernia utilising three-point stomal pexy, retrorectus circumstomal mesh placement, and lateral transversus abdominis release using a lateral crescentic incision.

## 2. Case Presentation

This case report and review of the literature was conducted according to the SCARE consensus statement and CARE guidelines (Document S1) [1]. The patient provided informed consent for their de-identified medical information to be used. Herein, we report the case of an 81-year-old female who underwent a lateral approach circumstomal open retrorectus mesh repair with transversus abdominis release and three-point stomal pexy for an acute-on-chronic complex parastomal hernia causing high-grade gastric outlet obstruction.

### 2.1. History and Examination

An 81-year-old female presented to the emergency department with five episodes of acute vomiting, abdominal distension and reduced stoma output to 100 mL over the prior 48 h. She denied any urinary symptoms, weight loss, recent illness, dysphagia or any issues with her stoma leading up to this presentation. This occurred against the background of a hospital presentation for abdominal pain four weeks prior, with a work-up including a computed tomography (CT) scan of the abdomen and pelvis that demonstrated a large, uncomplicated, left-sided parastomal hernia containing small bowel and colon (Figure 1A,B). She was discharged home the same day without intervention. Her past history was significant for a Hartmann’s procedure (proctosigmoidectomy with formation of an end colostomy) in 2021 for acute perforated sigmoid diverticulitis, a known chronic parastomal hernia over the last four years, chronic obstructive pulmonary disease (COPD) on Symbicort, and rheumatoid arthritis that was well managed with prednisolone, methotrexate and hydroxychloroquine without any complications. Of note, she reported no acute flares of her COPD or rheumatoid arthritis, as well as no recent hospitalisations due to these comorbidities. Functionally, she was independent with personal hygiene, mobilised with a four-wheeled walker and required assistance with community activities of daily living. She denies smoking and alcohol consumption. On examination, the patient’s vital signs were all within normal limits. Her abdomen was soft and was significant for a large, soft and reducible parastomal hernia over the site of her left lower quadrant end colostomy.

### 2.2. Differential Diagnosis and Investigations

Initial concerns for this patient were for an adhesional small bowel obstruction, particularly given the chronicity of her parastomal hernia, which had never caused issues. Biochemical investigations were significant for a haemoglobin of 89 g/L (chronic in the setting of anaemia of chronic disease secondary to the patient’s comorbidities), a white cell count (WCC) of 14.2 × 10^9^/L, a C-reactive protein (CRP) level of 10 mg/L and a lactate of 1.0 mmol/L. A CT scan of the abdomen and pelvis demonstrated an acute-on-chronic parastomal hernia with a neck width of approximately 4.7 cm that had previously been identified; however, it now contained a distended stomach being pulled into the left lower quadrant. This presumably caused tight angulation and kinking of the gastroduodenal junction where the pylorus transitions into the duodenum, and subsequent high-grade mechanical gastric outlet obstruction (Figure 2). A nasogastric tube was inserted for decompression and immediately drained 1.5 L of gastric content.

### 2.3. Management

The patient was fasted and proceeded to surgical repair of the parastomal hernia given concerns for ongoing gastric outlet obstruction and potential hernia complications such as strangulation if no repair was performed. Importantly, this decision was made following discussion with the patient and her family, given that the patient was relatively optimised from a comorbidity perspective, wanted to maintain independence, wanted to avoid further nasogastric tube insertion in the event of recurrence, wanted to avoid further pain and hospital presentations and had already recently presented with an acute parastomal hernia in the prior weeks. An open approach was adopted given the acute emergency setting, obstruction and the need to directly visualise the closure of each layer of the defect to achieve definitive closure. She was placed supine, and once under general anaesthesia with full muscle relaxation, the hernia was reduced. Crescentic left lateral skin markings to delineate the site of the incision were drawn (Figure 3). Following standard preparation and draping, a sterile, iodine-impregnated adhesive incise drape was placed over the abdomen to ensure that the stoma bag would not contaminate the operative field. An incision was made over the skin markings, and dissection was made to the anterior sheath. The hernia sac was mobilised off the anterior sheath to delineate the margins of the hernia defect, which was approximately 4.0 cm by 4.5 cm, corresponding well with imaging findings. The sac at this point mainly comprised small bowel, as the stomach was reduced following a combination of nasogastric decompression, full muscle relaxation and manual reduction following induction. Therefore, the decision was made to enter the peritoneal cavity to safely reduce the small bowel into the abdomen. The stomach was examined, and no evidence of ischaemia was identified. The chronic sac adhesions to the stoma were also divided. Stomal resitting was considered; however, it was avoided to minimise operative time, further morbidity and the risk of further recurrence at the site of a resited stoma and the old hernia site due to the patient’s comorbidities. Accordingly, a circumstomal retrorectus plane was developed. A stoma pexy was then performed with 2-0 Vicryl interrupted sutures to the left lateral abdominal wall. The peritoneum was then plicated with 2-0 Vicryl linearly and also circumstomally with 2-0 Vicryl to form an airtight peritoneal seal (not pictured). A transversus abdominis release was performed laterally to achieve complete tension-free closure of the posterior layers around the stoma aperture, minimising the structural risk of early failure. Using an 8 cm Ventralex circular mesh, a 30 mm circle was drawn and cut in the centre to create a “donut”, and then a radial incision was made in the mesh to create an opening to allow the mesh to encircle the stoma with additional overlap of approximately 2 cm. The mesh was then impregnated with a gentamicin wash, placed in the retrorectus plane, and fixed with 2-0 Prolene sutures to the posterior rectus sheath, deep to the rectus muscle (Figure 4). The mesh was placed intentionally with the bioresorbable hydrogel barrier facing posteriorly and resting on the posterior sheath/peritoneum and the polypropylene surface oriented anteriorly in direct contact with the rectus muscle. Placement in this way separated the mesh from the peritoneum and abdominal cavity, thereby decreasing the risk of adhesion formation or erosion. Furthermore, this placement also allowed direct contact of the polypropylene mesh surface with the rectus muscle, facilitating weaving and integration of the mesh with the muscle to prevent mesh movement and to improve structural integrity of the repair. These factors are also the reason behind this choice of mesh, rather than a conventional flat mesh. The anterior sheath was then closed with 1-0 nylon interrupted sutures with a pexy of the stoma circumferentially to the anterior sheath with 2-0 Vicryl, representing a three-point stomal pexy (Figure 5). A 15 French Blake’s drain was placed in the subcutaneous tissue, which was then closed sequentially with 2-0 Vicryl. The skin was closed with Monocryl (Figure 6). The stoma was checked at the end of the case and showed no signs of ischaemia.

### 2.4. Outcome and Follow-Up

The patient recovered well on the ward with routine escalation of her diet. The drain was kept in with oral antibiotic cover until it reached an output of <30 mL/24 h, which occurred on day 6 of admission. She was ready for discharge within a week of the parastomal hernia repair; however, she sustained a spontaneous, atraumatic index finger extensor tendon rupture due to her long-term treatment with steroids and extremely poor tissue quality, preventing discharge as she was unable to care for her stoma with the impairment of her hand function, therefore delaying her discharge. She was seen by the plastic surgery team, who opted for semi-acute repair of the tendon, and so was discharged home two weeks following the procedure with no issues otherwise. She had no evidence of recurrence at the four-week mark.

## 3. Discussion

Parastomal hernia has been estimated to occur with an incidence ranging from 0 to up to 50% [2]. Key determinants for this variable presentation include the type of stoma created, in addition to patient factors including obesity, female gender and age over 60 years (which may relate to the laxity of tissues) and the presence of disorders that may predispose patients to increased intra-abdominal pressure (such as COPD) [3,4]. Reports from the European Hernia Society suggest that the majority of parastomal hernias contain omentum, small intestine or colon and typically develop years after the initial creation of the stoma [5,6]. Stomach herniation into a parastomal hernia is exceedingly rare, with 13 cases reported in 2018 and 20 cases reported in the literature by 2022 [7,8]. This is in part due to the anatomical location of the stomach, which is surrounded and fixed by local structures including the diaphragm, duodenum, spleen and transverse colon. Despite this, risk factors as noted for this case, including age, female gender, COPD, immunosuppression and obesity, predispose this patient to potential increased abdominal pressure, enlargement of the fascial defect and tissue laxity that culminate in the development of the gastric parastomal hernia with potential incomplete or complete gastric outlet obstruction. Furthermore, for this patient, it is likely that the first episodes of obstruction were due to small bowel and colonic obstruction. In the presence of adhesions between the bowel and sac, it is possible there was no space for gastric herniation.

As for this case and many described in the literature, following diagnostic work-up, including a CT scan of the abdomen and pelvis to define the anatomy and characterise the degree of obstruction, insertion of a nasogastric tube should be considered in the first instance for decompression and to assist with reduction and, therefore, management of the acute obstruction and/or incarceration [7,9]. Following this, repair of the parastomal hernia should be considered; however, the best approach to repair, including timing and approach, remains an area of debate. In a large national cohort study of over 7000 patients, Howard et al. reported that recurrence outcomes were lowest with reversal of the stoma [10]. The authors also noted that in those for whom reversal was not an option, repair with or without stomal resitting was relatively equivalent in terms of reoperation rate, although this also included a population with recurrence that did not require an operation [10]. Importantly, the cohort studied was in the elective setting, a particularly relevant consideration, as other studies have reported acute repair of parastomal hernias to be the strongest risk factor for reoperation [11]. Additionally, Howard et al. also reported variably on the method of hernia repair, such as the location of mesh placement and type of closure, which are additional confounding variables; therefore, the results may not necessarily reflect what would be observed for a patient with an emergently presenting acute gastric parastomal hernia. Nonetheless, when it comes to the surgical approach, a systematic review by Hansson et al. identified superior outcomes with the laparoscopic Sugarbaker method as well as with mesh [12].

Despite the evidence related to improved recurrence rates with elective and minimally invasive repair approaches, our case was challenged by the potential for repeated gastric outlet obstruction in a patient who had comorbidities but was optimised and expressing a desire to remain independent and be able to cope at home. The decision was therefore made to undertake an acute repair approach. Furthermore, we report the first case detailing a lateral circumstomal open retrorectus mesh repair with transversus abdominis release and three-point stomal pexy for the repair of a large parastomal hernia repair containing the stomach. The choice of mesh placement in this case was inspired by the evidence that highlights retrorectus repair of ventral hernias as the preferred standard when it comes to recurrence outcomes [13]. Despite this, the authors examine this in the context of a midline non-complex ventral hernia, a population different from the patient encountered in this case. Furthermore, the evidence suggests that the larger the hernia defect aperture, the greater the chance of hernia formation and recurrence [14]. To reduce the chances of further recurrence, we adopted a three-point fixation of the stoma, including lateralisation to the left lateral abdominal wall to represent a Sugarbaker configuration, as well as stomal pexy with sutures to the posterior sheath and anterior sheath, together with a transversus abdominis release to maximise tension-free closure [14]. The approach adopted in this case was therefore conceptually guided by data regarding the management of elective midline non-complex ventral hernias as well as minimally invasive approaches for elective parastomal hernia repair. Consequently, it must be acknowledged that extrapolating these outcomes to a complex, lateral parastomal hernia is a limitation.

The question of whether an open or a laparoscopic approach should be used is another consideration that is debated. At present, there is a lack of evidence that suggests one approach is superior with respect to recurrence outcomes, although it is noted that a laparoscopic approach may be associated with improved length of stay and superficial surgical site infection outcomes [14]. The choice of an open procedure in this case was based on multiple reasons, including surgeon preference and patient/family discussions, as well as the opportunity to visualise the defect, allowing for more meticulous closure of the hernia defect layers.

## 4. Conclusions

This case highlights and provides additional insight into the rare presentation of a gastric parastomal hernia. We describe a novel approach to repair, including a lateral incision, circumstomal open retrorectus mesh repair with transversus abdominis release, and three-point stomal pexy as an alternative method to address acute repair in a patient with comorbidities with the potential for recurrent mechanical gastric outlet obstruction. Longer prospective follow-up is required to evaluate the durability and recurrence outcomes of this approach.

## Figures and Tables

**Figure 1:**
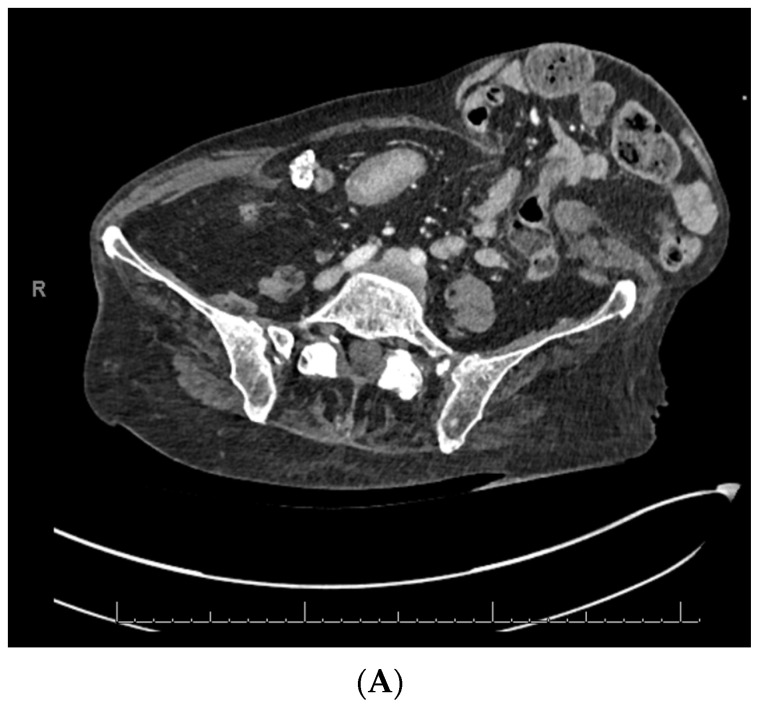

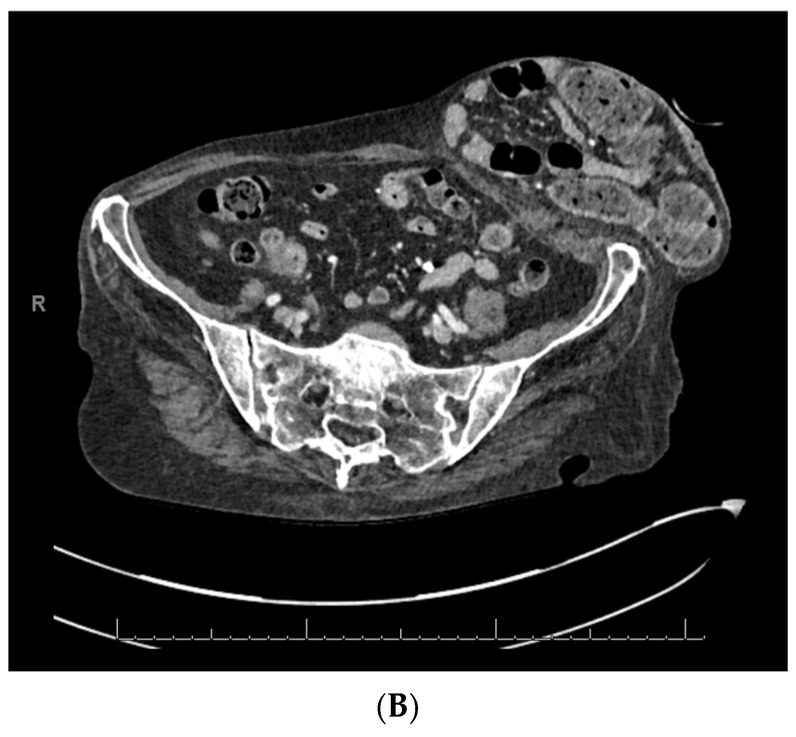
(**A**) Axial CT of the abdomen and pelvis demonstrating an uncomplicated parastomal hernia with a wide neck containing small bowel and colon. (**B**) Axial CT of the abdomen and pelvis demonstrating an uncomplicated parastomal hernia more inferiorly.

**Figure 2:**
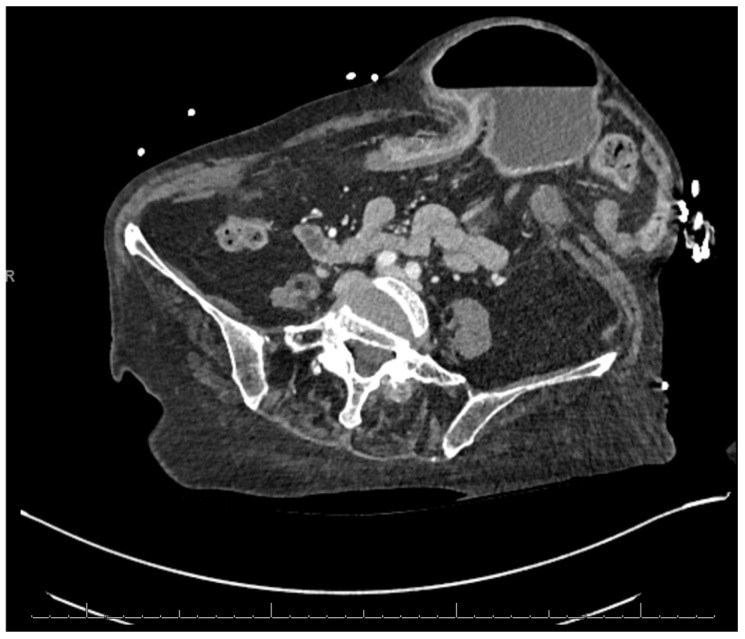
CT scan of the abdomen and pelvis demonstrating a parastomal hernia now containing a distended stomach causing tight angulation of the gastroduodenal junction and subsequent high-grade mechanical gastric outlet obstruction.

**Figure 3:**
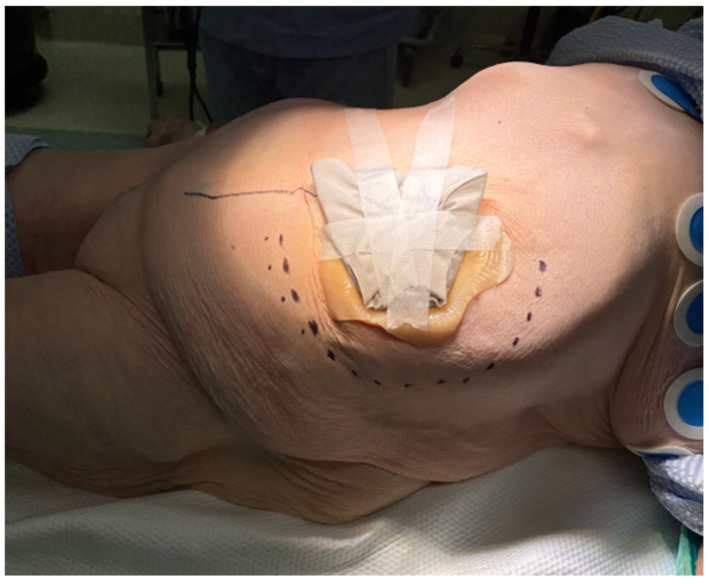
Left lateral crescentic skin marking performed prior to incision.

**Figure 4:**
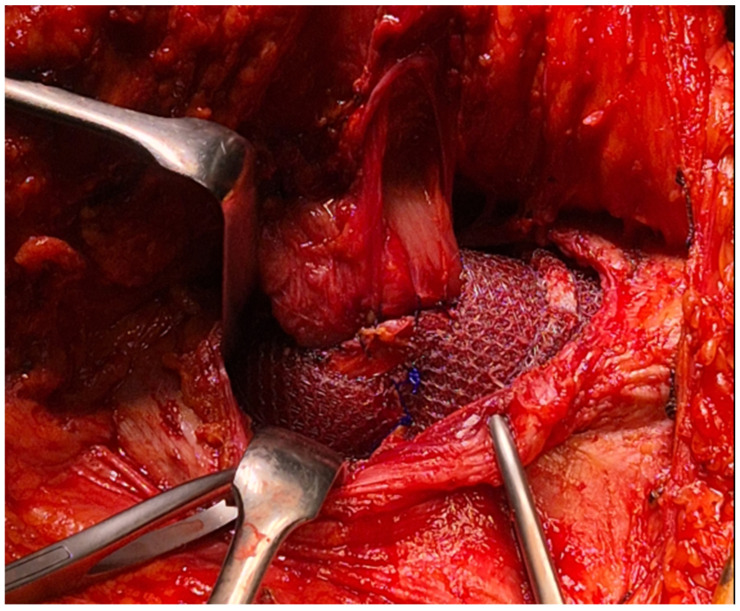
Clinical intra-operative photograph demonstrating placement of an 8 cm Ventralex circular mesh that was cut to form a donut shape, impregnated with a gentamicin wash and placed in the retrorectus plane around the stoma.

**Figure 5:**
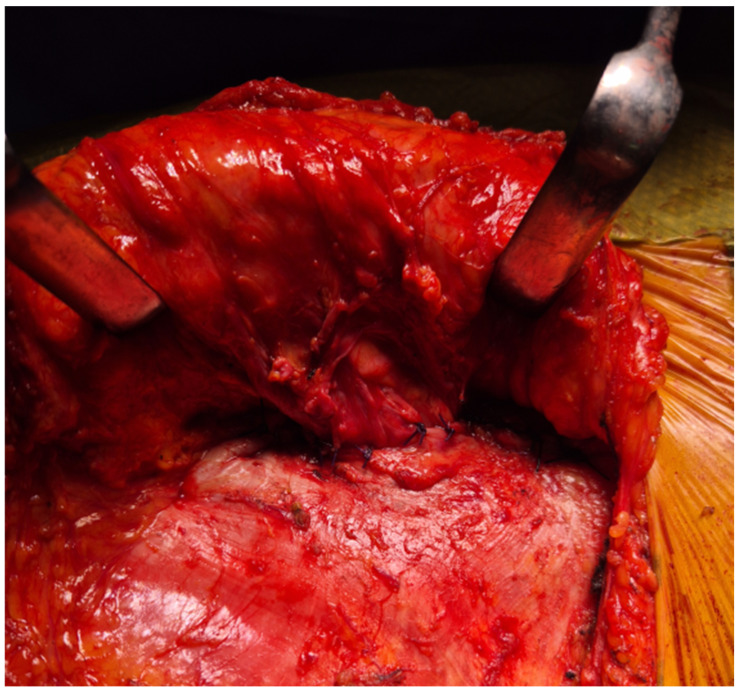
Clinical intra-operative photograph demonstrating closure of the anterior sheath with nylon and circumstomal pexy of the anterior sheath with 2-0 Vicryl.

**Figure 6:**
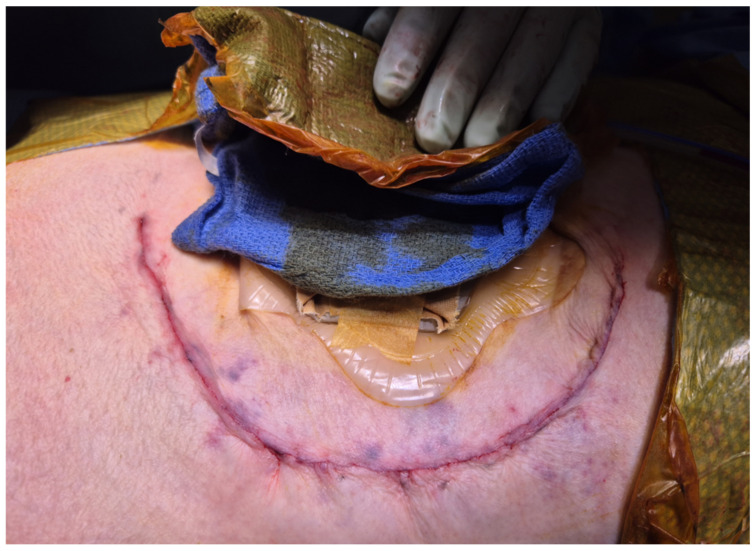
Clinical intra-operative photograph of skin closure.

## Data Availability

Data can be requested from corresponding author when required. All relevant data have been provided in the generation of this manuscript, which is intended for open access publication.

## Supplementary Materials

The following supporting information can be downloaded at: https://www.mdpi.com/article/10.3390/jcm15135193/s1, Document S1: CARE Checklist of information to include when writing a case report.
