# Supplementary material for: Lateral Approach Circumstomal Open Retrorectus Mesh Repair with Transversus Abdominis Release and Three-Point Stomal Pexy for an Acute-on-Chronic Complex Parastomal Hernia Causing High-Grade Gastric Outlet Obstruction: A Case Report
Source: J Clin Med. 2026 Jul 2;15(13):5193. doi: 10.3390/jcm15135193 (PMC13363675; doi:10.3390/jcm15135193)
Supplement: Supplementary file 1 [file jcm-15-05193-s001.zip › jcm-4396869-supplementary.pdf]

CARE case report guidelines

CARE Checklist of information to include when writing a case report

(cc) BY-NC-ND

Topic	Item No	Checklist item description	Reported on Page Number/Line Number	Reported on Section/Paragraph
Title	1	The diagnosis or intervention of primary focus followed by the words "case report"	Line 3-5	Page 1
Key Words	2	2 to 5 key words that identify diagnoses or interventions in this case report, including "case report"	Line 72	Page 3
Abstract (Structured summary)	3a	Background: state what is known and unknown; why the case report is unique and what it adds to existing literature.	Line 63-70	Page 3
	3b	Case Description: describe the patient's demographic details, main symptoms, history, important clinical findings, the main diagnosis, interventions, outcomes and follow-ups.	Line 63-70	Page 3
	3c	Conclusions: summarize the main take-away lesson, clinical impact and potential implications.	Line 63-70	Page 3
Introduction	4	One or two paragraphs summarizing why this case is unique **(may include references)**	Line 83-88	Page 4
Patient Information	5a	De-identified patient specific information	Line 81-111	Page 4-5
	5b	Primary concerns and symptoms of the patient	Line 81-111	Page 4-5
	5c	Medical, family, and psycho-social history including relevant genetic information	Line 81-111	Page 4-5
	5d	Relevant past interventions with outcomes	Line 81-111	Page 4-5
Clinical Findings	6	Describe significant physical examination (PE) and important clinical findings	Line 110-120	Page 5
Timeline	7	Historical and current information from this episode of care organized as a timeline	Line 81-111	Page 4-5
Diagnostic Assessment	8a	Diagnostic testing (such as PE, laboratory testing, imaging, surveys).	Line 113-120	Page 5
	8b	Diagnostic challenges (such as access to testing, financial, or cultural)	Line 110-120	Page 5
	8c	Diagnosis (including other diagnoses considered)	Line 110-120	Page 5
	8d	Prognosis (such as staging in oncology) where applicable	N/A	N/A
Therapeutic Intervention	9a	Types of therapeutic intervention (such as pharmacologic, surgical, preventive, self-care)	Line 122-149	Page 5-6
	9b	Administration of therapeutic intervention (such as dosage, strength, duration)	Line 122-149	Page 5-6
	9c	Changes in therapeutic intervention (with rationale)	Line 122-149	Page 56

Follow-up and Outcomes	10a	Clinician and patient-assessed outcomes (if available)	Line 151-158	Page 6
	10b	Important follow-up diagnostic and other test results	Line 151-158	Page 6
	10c	Intervention adherence and tolerability (How was this assessed?)	N/A	N/A
	10d	Adverse and unanticipated events	Line 151-158	Page 6
Discussion	11a	A scientific discussion of the strengths AND limitations associated with this case report	Line160-216	Page 7-8
	11b	Discussion of the relevant medical literature **with references**	Line160-216	Page 7-8
	11c	The scientific rationale for any conclusions (including assessment of possible causes)	Line160-216	Page 7-8
	11d	The primary "take-away" lessons of this case report (without references) in a one paragraph conclusion	Line 218-223	Page 8-9
Patient Perspective	12	The patient should share their perspective in one to two paragraphs on the treatment(s) they received	N/A	N/A
Informed Consent	13	Did the patient give informed consent? Please provide if requested	**Yes** ☐	☐

Please leave this space alone as it will be supplemented by the editorial office when needed.
